# Supplementary material for: Staff perspectives on fall prevention activities in long-term care facilities for older residents: "Brief but often" staff education is key
Source: PLoS One. 2024 Sep 9;19(9):e0310139. doi: 10.1371/journal.pone.0310139 (PMC11383231; doi:10.1371/journal.pone.0310139)
Supplement: S1 File — (DOCX) [file pone.0310139.s001.docx]

Welcome. Thank you for coming along today and for giving your time to take part in this focus group. We appreciate your willingness to contribute and are interested to hear your views. My name is **NA** and I work in the Centre for Gerontology and Rehabilitation at UCC. I will moderate today’s discussion.

**Agenda review:**

1. Purpose of meeting
2. Few words about audio-recording
3. Ground rules

4. Brief round of introductions

5. Begin discussing the interview questions

1. **Purpose of the interviws :** As outlined in previous emails, we are trying to understand more about the barriers to preventing falls among residents, along with staff solutions and opinions for overcoming barriers. You have been invited here because you can speak directly from personal experience. We need your input and want you to share your thoughts with us in an open and honest manner.

2. **Audio-recording:** We will be recording this session. We want to make sure we fully capture everyone’s contribution. This recording is for me to type out the dialogue, and it will be deleted after two weeks of this discussion.

No one will be identified by name in any report we produce. No real names or other direct information are reported.

Does everyone understand this, and is everyone okay with this recording?

3. **Ground Rules:**

a. I will endeavour to cover as many of these questions as possible to allow you to share your views on as many aspects as possible. There are no right or wrong answers, only differing points of view. We are interested in capturing as broad a range of views and experiences as possible, so you are encouraged to share whether you agree or disagree with what has already been said.

b.What is said in the room should stay here. We want everyone to feel comfortable to share their views fully and frankly.

1. When someone speaks, please use the mute button, and open it when you want to speak. Keep your speech clear so that everyone can catch what you are saying
2. Any questions before we begin?
3. **Introductions:** We will operate on a first-name basis. Let’s go around the group to introduce ourselves. Please just let us know your first name, and if you wish to say a few words about yourself and your interest in the subject matter of this focus group, please do (if not, that is fine too). Who wants to start?

**5. Guiding Questions**:

1. Do you believe falls in residents in your facility can be prevented? Why do you say this?
2. What makes it **harder** for you? to prevent residents’ falls/ reduce residents’ risk of falls?
3. For each barrier mentioned -How does it do this? How difficult is this to overcome? What could achieve this? for each of those barriers, what do you think is a solution
4. Based on your experience, what would **help you in your role** to reduce falls or risk or fall?
5. For each facilitator mentioned - How does this work? Have there been challenges with it? Are there any unintended consequences? Would this work on your site? What would be needed to implement it?
6. **What else** would help to reduce falls? Why? What has worked in other places that you have been employed e.g. acute care or another nursing home?
7. Have you attended a fall prevention education programme/session, if so, tell us about it. What did you cover/learn? How did you apply it to your practice? What was good about it? What was not good about it?
8. If falls prevention **training** was to be offered, would you sign up? Why/why not?
9. Have you any other comments or suggestions to help reduce residents’ falls?

**Conclusion**: Thank you very much for participating in this discussion. Your thoughts and opinions will be extremely valuable to the study. We hope you have found the discussion to be interesting and engaging. I would like to remind you that all of your comments will be anonymous, but it is also important to respect what others have shared today and not discuss it outside this group.

If you are uncomfortable with anything that came up in this discussion, please contact me directly following this interview.

Thank you, again, for your time today.
