# Supplementary material for: Staff perspectives on fall prevention activities in long-term care facilities for older residents: "Brief but often" staff education is key
Source: PLoS One. 2024 Sep 9;19(9):e0310139. doi: 10.1371/journal.pone.0310139 (PMC11383231; doi:10.1371/journal.pone.0310139)
Supplement: Attachment — Submitted filename: Response to Reviewers.docx [file pone.0310139.s002.docx]

Response to Reviewers

When submitting your revision, we need you to address these additional requirements.

1. Please ensure that your manuscript meets PLOS ONE's style requirements, including those for file naming. The PLOS ONE style templates can be found at
<https://journals.plos.org/plosone/s/file?id=wjVg/PLOSOne_formatting_sample_main_body.pdf> and
<https://journals.plos.org/plosone/s/file?id=ba62/PLOSOne_formatting_sample_title_authors_affiliations.pdf>

Response: Thank you for reviewing our manuscript. We have formatted all headings in the manuscript, from the abstract to the supporting information section after the reference list, according to the journal's guidelines. This includes using Heading 1, 2, 3, and so on, as specified by the formatting rules. In the results section, heading level 4 has also been included for all subthemes so as to reduce the use of italics. Additionally, we have adjusted the formatting of the manuscript’s title to comply with the journal's style requirements. Across the entire text, all changes are highlighted in red.

2. Note from Emily Chenette, Editor in Chief of PLOS ONE, and Iain Hrynaszkiewicz, Director of Open Research Solutions at PLOS: Did you know that depositing data in a repository is associated with up to a 25% citation advantage (<https://doi.org/10.1371/journal.pone.0230416>)? If you’ve not already done so, consider depositing your raw data in a repository to ensure your work is read, appreciated and cited by the largest possible audience. You’ll also earn an Accessible Data icon on your published paper if you deposit your data in any participating repository (<https://plos.org/open-science/open-data/#accessible-data>).

Response: Thank you for your note and for highlighting the potential citation advantage of depositing data in a repository. However, our data are qualitative and involve sensitive information and we must follow the policy of our research ethics board in UCC.

3. In the online submission form, you indicated that [Anonymous data used and analysed during this study are available from the corresponding author for non-commercial projects on reasonable request.]. 
All PLOS journals now require all data underlying the findings described in their manuscript to be freely available to other researchers, either 1. In a public repository, 2. Within the manuscript itself, or 3. Uploaded as supplementary information.
This policy applies to all data except where public deposition would breach compliance with the protocol approved by your research ethics board. If your data cannot be made publicly available for ethical or legal reasons (e.g., public availability would compromise patient privacy), please explain your reasons on resubmission and your exemption request will be escalated for approval.

Response: As stated above, due to the nature of the data, we are happy to provide data to others on a case-by-case basis, where we can limit or redact data as needed, to maintain confidentiality. However, we cannot have the full data set publicly available, as participants could be identified by the context of their interview responses (even though data is anonymised), which may include some overt criticism of the health service or local management structures. Participants consented to participation and were very honest in their responses based on the data being shared as we described in the PIL, and our ethics committee approved this protocol, thus we cannot now change to deposition in a repository, or inclusion within the main body or appendices of the paper, as this would be a breach of our ethics approval and participant trust.

4. Please include captions for your Supporting Information files at the end of your manuscript, and update any in-text citations to match accordingly. Please see our Supporting Information guidelines for more information: <http://journals.plos.org/plosone/s/supporting-information>.

Response: The supporting information has been added at the end of the manuscript, after the reference list (lines 946-947). On page 7, line 118, of our manuscript, we have updated that information.

5. Please review your reference list to ensure that it is complete and correct. If you have cited papers that have been retracted, please include the rationale for doing so in the manuscript text, or remove these references and replace them with relevant current references. Any changes to the reference list should be mentioned in the rebuttal letter that accompanies your revised manuscript. If you need to cite a retracted article, indicate the article’s retracted status in the References list and also include a citation and full reference for the retraction notice.

Response: We have checked all of the references, and no papers have been retracted. We have removed all of the URLs and we have included links to web pages or resources from national government websites.

Reviewers' comments:

Reviewer's Responses to Questions

**Comments to the Author**

1. Is the manuscript technically sound, and do the data support the conclusions?

The manuscript must describe a technically sound piece of scientific research with data that supports the conclusions. Experiments must have been conducted rigorously, with appropriate controls, replication, and sample sizes. The conclusions must be drawn appropriately based on the data presented.

Reviewer #1: Yes

2. Has the statistical analysis been performed appropriately and rigorously?

Reviewer #1: N/A

3. Have the authors made all data underlying the findings in their manuscript fully available?

The [PLOS Data policy](http://www.plosone.org/static/policies.action#sharing) requires authors to make all data underlying the findings described in their manuscript fully available without restriction, with rare exception (please refer to the Data Availability Statement in the manuscript PDF file). The data should be provided as part of the manuscript or its supporting information or deposited to a public repository. For example, in addition to summary statistics, the data points behind means, medians and variance measures should be available. If there are restrictions on publicly sharing data—e.g. participant privacy or use of data from a third party—those must be specified.

Reviewer #1: Yes

4. Is the manuscript presented in an intelligible fashion and written in standard English?

PLOS ONE does not copyedit accepted manuscripts, so the language in submitted articles must be clear, correct, and unambiguous. Any typographical or grammatical errors should be corrected at revision, so please note any specific errors here.

Reviewer #1: Yes

5. Review Comments to the Author

Please use the space provided to explain your answers to the questions above. You may also include additional comments for the author, including concerns about dual publication, research ethics, or publication ethics. (Please upload your review as an attachment if it exceeds 20,000 characters)

Reviewer #1: I think it is very well written. Please address the following minor points.

1. Lines 238-240 suggest that having experienced staff reduces the risk of falls. Specifically, what is the difference between younger and more experienced staff? I would like to see a few sentences in the discussion to include perspectives from previous studies.
Response: Thank you for bringing this to our attention. We have discussed this in detail on pages 31 and 32, lines 649-662.

2. line573
Topic 5 is probably a mistake for topic 6.

Response: Thank you for pointing this out. This has been corrected to “Theme 6” on page 28, line 573.
