# Supplementary material for: Staff perspectives on fall prevention activities in long-term care facilities for older residents: "Brief but often" staff education is key
Source: PLoS One. 2024 Sep 9;19(9):e0310139. doi: 10.1371/journal.pone.0310139 (PMC11383231; doi:10.1371/journal.pone.0310139)
Supplement: Attachment — Submitted filename: Response to Reviewers.docx [file pone.0310139.s003.docx]

Journal  Requirements:

Please review your reference list to ensure that it is complete and correct. If you have cited papers that have been retracted, please include the rationale for doing so in the manuscript text, or remove these references and replace them with relevant current references. Any changes to the reference list should be mentioned in the rebuttal letter that accompanies your revised manuscript. If you need to cite a retracted article, indicate the article’s retracted status in the References list and also include a citation and full reference for the retraction notice.

Response: We have checked all of the references, and no papers have been retracted. We have removed all of the URLs, and we have included links to web pages or resources from national government websites. We have reviewed and removed all the old papers from the reference list except those that we considered important to our paper.

[Note: HTML markup is below. Please do not edit.]

Reviewers' comments:

Reviewer's Responses to Questions

**Comments to the Author**

1. If the authors have adequately addressed your comments raised in a previous round of review and you feel that this manuscript is now acceptable for publication, you may indicate that here to bypass the “Comments to the Author” section, enter your conflict of interest statement in the “Confidential to Editor” section, and submit your "Accept" recommendation.

Reviewer #1: All comments have been addressed

Reviewer #2: (No Response)

2. Is the manuscript technically sound, and do the data support the conclusions?

The manuscript must describe a technically sound piece of scientific research with data that supports the conclusions. Experiments must have been conducted rigorously, with appropriate controls, replication, and sample sizes. The conclusions must be drawn appropriately based on the data presented.

Reviewer #1: Yes

Reviewer #2: Yes

3. Has the statistical analysis been performed appropriately and rigorously?

Reviewer #1: Yes

Reviewer #2: Yes

4. Have the authors made all data underlying the findings in their manuscript fully available?

The [PLOS Data policy](http://www.plosone.org/static/policies.action#sharing) requires authors to make all data underlying the findings described in their manuscript fully available without restriction, with rare exception (please refer to the Data Availability Statement in the manuscript PDF file). The data should be provided as part of the manuscript or its supporting information, or deposited to a public repository. For example, in addition to summary statistics, the data points behind means, medians and variance measures should be available. If there are restrictions on publicly sharing data—e.g. participant privacy or use of data from a third party—those must be specified.

Reviewer #1: Yes

Reviewer #2: Yes

5. Is the manuscript presented in an intelligible fashion and written in standard English?

PLOS ONE does not copyedit accepted manuscripts, so the language in submitted articles must be clear, correct, and unambiguous. Any typographical or grammatical errors should be corrected at revision, so please note any specific errors here.

Reviewer #1: Yes

Reviewer #2: Yes

6. Review Comments to the Author

Please use the space provided to explain your answers to the questions above. You may also include additional comments for the author, including concerns about dual publication, research ethics, or publication ethics. (Please upload your review as an attachment if it exceeds 20,000 characters)

Reviewer #1: The authors have responded appropriately and effectively to the comments.
No further revision required.

Reviewer #2: Clearly written and scholarly article. Only a few points need to be addressed:

1. The overall length of this manuscript is far too long - please reduce by at least 300 words.

Response: We have reduced the total word count of the text by around 650 words, as it originally contained 8,109 words, and now it has (7,461 words)

2. The title is excessively long and complication - I had to read it several times. Please do a major revision of the title to make it clear and concise rather than long and descriptive.

Response: We have revised the title to make it clearer

3. The methods section is excessively long and I am concerned that some sections on qualitative methods seem to be very similar to the original source - please run the manuscript thorough iThenticate (or similar) and reduce text so you are not repeating other authors words so much. Just because you give a reference does not mean that you can have large chucks of very similar text.

Response: We checked for any plagiarism and could not find any. We are confident that our manuscript is well-written and is free of plagiarism. Our methods section was written in depth, as we followed the COREQ guideline, and most of the items focused on the methods. In accordance with the guideline, we have reported all of our procedures in detail. However, we have acknowledged that our methods section was too long, as it contained 1,205 words, so we reduced it to (908 words).

4. The Discussion could cite and discuss the learnings from the hospital falls literature a bit more as these are highly relevant and would bring your review up to date. Some refs to consider include:

Morris, M.. (2024). Preventing hospital falls: feasibility of care workforce redesign to optimise patient falls education. Age and Ageing, 53(1), 9 pages. doi:10.1093/ageing/afad250
Montero-Odasso, M., Van Der Velde, N., Martin, F. C., Petrovic, M., Tan, M. P., Ryg, J., . . . Gac Espinola, H. (2022). World guidelines for falls prevention and management for older adults: a global initiative. Age and Ageing, 51(9). doi:10.1093/ageing/afac205
Morris, M. E.,(2022). Interventions to reduce falls in hospitals: a systematic review and meta-analysis.. Age and Ageing, 51(5), 12 pages. doi:10.1093/ageing/afac077
Heng, H.,. (2020). Hospital falls prevention with patient education: a scoping review. BMC Geriatrics, 20, 12

Response: Thank you for the recommendations you provided. The resources are beneficial since they offer significant knowledge on the prevention of falls among older people. However, our primary objective is to prevent falls among older people residing in long-term care facilities. Consequently, we will not include all these references, as they pertain to the context of acute care (hospitals) and are not relevant to our study. We have included the SR by Morris et al as a usual reference for readers. Also, we already had referenced the World Guidelines for Falls Prevention (the second reference from Montero-Odasso et al., as you suggested) (reference number 7 (previously 47) in our manuscripts).

5. Please update the ref list to include more recent refs.

Response: Our reference list has been reviewed and updated with a few new papers, and older papers have been removed except those that are important for comparing our results for context.
